# Announcing the establishment of new speciality section on quantum biology and biophotonics

**DOI:** 10.1016/j.csbj.2023.12.013

**Published:** 2023-12-14

**Authors:** Youngchan Kim

**Affiliations:** a Department of Microbial Sciences, School of Biosciences, University of Surrey, Guildford GU2 7XH, UK; b Leverhulme Quantum Biology Doctoral Training Centre, University of Surrey, Guildford GU2 7XH, UK; c Advanced Technology Institute, University of Surrey, Guildford GU2 7XH, UK

We are thrilled to announce the establishment of the new speciality section on Quantum Biology and Biophotonics within the Computational and Structural Biotechnology Journal (CSBJ). This speciality section advocates for a transdisciplinary approach that integrates diverse perspectives to unravel the intricate mechanisms underpinning the interplay between quantum phenomena and biological systems. Furthermore, this section focuses on tremendous innovations in optics and photonic tools, methods, and technologies to address questions in quantum biology and its potential impact on biotechnology, biomedicine, and healthcare.

Quantum Biology has emerged as a vibrant interdisciplinary research field, propelled by recent advancements in physics, biology, and chemistry. It posits that quantum mechanics play a pivotal role in diverse biological processes, including animal navigation, olfactory sensing, vision, cellular metabolic regulation, cell physiology, photosynthesis, quantum tunnelling in enzymes and DNA mutation, electron-spin dependent chemical reactions in biology, bioelectronics, ion channel functions in neurobiology, and more. This is an exciting and emerging scientific exploration, paving the way for innovative discoveries and developments that will revolutionize healthcare. Integrating observations of quantum biology phenomena across various length and time scales remains a significant challenge. Additionally, language and methodology barriers hinder community building efforts. The success of quantum biology endeavours hinges on interdisciplinarity, multi-scale approaches, and close collaboration between theory and experiment, attainable only within a robust research collaboration network.

On the other hand, quantum technology has ushered in an unprecedented era of sensitivity and capability in physical measurements, harnessing the principles of quantum mechanics to transcend the limitations insurmountable by classical physics. Despite these remarkable achievements, translating these quantum technology breakthroughs into practical applications in biosciences and medicine remains a formidable challenging.

This new specialized section on quantum biology and biophotonics will serve as a dedicated platform for disseminating and discussing cutting-edge research in the field of quantum biology and biophotonics, fostering communication links among the currently siloed scientists worldwide.

We invite you to join these endeavours and establish this prominent and high-visibility open-access forum dedicated to quantum biology and biophotonics.

